# The prognostic value of two histopathologic classification models of ANCA-associated glomerulonephritis: a prospective study

**DOI:** 10.1007/s40620-023-01855-x

**Published:** 2024-02-12

**Authors:** Michalis Christodoulou, Eleni Moysidou, Georgios Lioulios, Stamatia Stai, Konstantinos Bandis, Nikolaos Flaris, Christina Nikolaidou, Asimina Fylaktou, Aikaterini Papagianni, Maria Stangou

**Affiliations:** 1 School of Medicine, Department of Nephrology, Aristotle University of Thessaloniki, Hippokration Hospital, Konstantinoupoleos 49, 54642 Thessaloniki, Greece; 2 Department of Pathology, Hippokration General Hospital, 54642 Thessaloniki, Greece; 3 Department of Immunology, National Histocompatibility Center, Hippokration General Hospital, 54642 Thessaloniki, Greece

**Keywords:** ANCA, Glomerulonephritis, Vasculitis, Biopsy, Berden, Renal risk score

## Abstract

**Background:**

Berden Classification and anti-neutrophil cytoplasmic antibody (ANCA) Renal Risk Score are classification models for rating renal histology and predicting outcome in patients with ANCA-associated Vasculitis/Glomerulonephritis (AAV/GN). In the present study we compare their ability to predict renal function outcome in short- and long-term follow up.

**Methods:**

Patients with an initial diagnosis of AAV/GN based on kidney biopsy were classified according to Berden and Renal Risk Score, started on the same treatment protocol, and were followed prospectively for up to 60 months. Renal function was recorded at 3mo(T3), 6mo(T6) and 60mo(T60), and results were compared to both classification systems.

**Results:**

Ninety four AAV/GN patients, M/F = 36/58, age = 60.05 (18–82)yrs were included. Based on Berden classification, patients grouped as Focal (*n* = 24), Crescentic (*n* = 35), Mixed (*n* = 21) and Sclerotic (*n* = 14), had significant differences in estimated glomerular filtration rate (eGFR) only at T3, while the percentage of those requiring hemodialysis differed at T0, T3, T6 but not at T60. According to the Renal Risk Score, patients were classified as Low (*n* = 8), Medium (*n* = 47) and High (*n* = 39) risk, and showed significant differences in both eGFR levels, proportion of hemodialysis, at T0, T3, T6 and end-stage kidney disease (ESKD) at T60. Even patients classified as Mixed (Berden) and as Medium or High risk (Renal Risk Score) had significant improvement from T0 to T6. Relapse could not be predicted by either system.

**Conclusion:**

Both methods were able to predict short-term renal function outcome and need for hemodialysis, but the Renal Risk Score showed significant superiority in predicting renal function outcome and ESKD after long-term follow up.

**Graphical Abstract:**

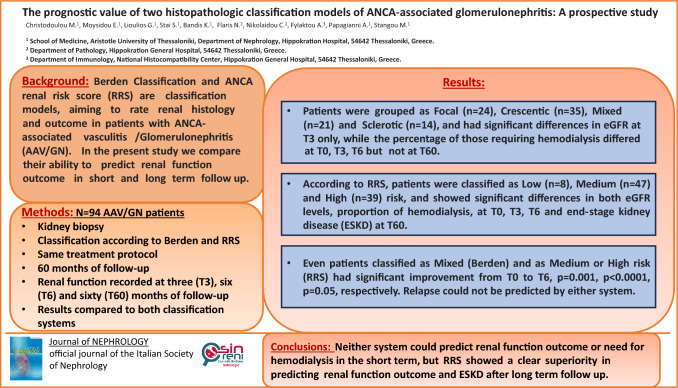

**Supplementary Information:**

The online version contains supplementary material available at 10.1007/s40620-023-01855-x.

## Introduction

ANCA-associated vasculitides (AAV) represent a group of systemic diseases characterized by inflammation and necrosis of small or medium-sized blood vessels. This entity includes granulomatosis with polyangiitis, eosinophilic granulomatosis with polyangiitis, and microscopic polyangiitis [1] and is accompanied by the presence of anti-neutrophil cytoplasmic antibodies (ANCA) in the serum [1–4].

The proposed mechanism by which ANCA are involved in the pathogenesis of necrotizing vasculitis includes the activation of neutrophils, inflammatory cytokines, the release of reactive oxygen species and enzymes, and the formation of neutrophil extracellular traps [2–4]. Renal involvement is undoubtedly one of the most important manifestations, as it determines both the treatment approach and outcome. In clinical practice, ANCA-associated glomerulonephritis (GN) starts as an acute, urgent situation that needs immediate diagnosis and treatment, and ends up as a chronic progressive disease that needs treatment modification, long term follow up and prevention from relapses.

Renal lesions, most frequently seen in ANCA vasculitis, include focal and segmental glomerular fibrinoid necrosis and crescent formation, while tubulointerstitial inflammation and fibrosis, and small vessel necrotizing vasculitis are also present. Although glomerular lesions progress rapidly and evolve to secondary focal or global glomerulosclerosis, there is usually still a number of unaffected, normal glomeruli which retain their architecture and seem to play an important role in the outcome of renal function.

Several classification systems have been described, based on either clinical, histological findings, or both, that are adopted to place patients in groups most likely to benefit from immunosuppression [5–9]. Since 2010, histopathologic classification models have been used to predict the renal outcome in patients with ANCA-associated GN, the most popular being the Berden Classification and ANCA Renal Risk Score [5–7]. Both classifications recognize and emphasize the importance of the remaining normal glomeruli.

“Berden classification” proposes four classes including: focal (≥ 50% normal < 50% injured glomeruli), crescentic (≥ 50% active crescents), mixed (≥ 50% injured glomeruli, < 50% crescents, < 50% global sclerosis) and sclerotic (≥ 50% global sclerosis) [8]. A multicenter study has shown that renal survival was much higher in the focal class (93%) than in the sclerotic (50%) class. The results regarding crescentic and mixed class were similar but inconclusive [8, 9].

The ANCA “Renal Risk Score” is calculated according to the percentage of normal glomeruli (0 points if > 25%, 4 points if 10–25%, 6 points if < 10%), the percentage of interstitial fibrosis and tubular atrophy (IFTA: 0 points if ≤ 25%, 2 points if > 25%) and the estimated glomerular filtration rate (eGFR) at diagnosis (0 points if > 15 ml/min, 3 points if ≤ 15 ml/min). According to the total Renal Risk Score, patients with 0 points are classified as low, 2–7 points as medium, and 8–11 points as high risk groups [10, 11].

Prognosis of patients with AAV has improved throughout the years but the risk of developing end-stage kidney disease (ESKD) remains high. Histopathologic lesions can provide useful information about the possibility of ESKD development in those patients. Only a few studies comparing these models exist, and yet, there are not sufficient data about which model is superior regarding specific factors that can determine renal outcome [11].

In the present study, we assessed the two most popular classification systems, the Berden and the Renal Risk Score, and evaluated their efficacy in predicting renal function outcome in patients with ANCA-associated GN, in short- and long-term follow up.

## Patients and methods

### Patients

In the present prospective observational study, we recruited patients with a first diagnosis of ANCA-associated GN. Diagnosis was always based on kidney biopsy findings, performed before commencing induction treatment. Patients’ demographic data, medical history, comorbidities, type of diagnosis, clinical manifestations and biochemical parameters were recorded at the time of kidney biopsy, regarded as the time of diagnosis (T0). All patients received the same treatment protocol, as described below, they had a regular follow up in the Vasculitis clinic, and their renal function was recorded at time points 3 (T3), 6 (T6) and 60 (T60) months during follow-up.

### Inclusion criteria

- Age > 18 years old.
- Patients with histopathologically-proven ANCA-associated GN (AAV/GN).
- Minimum of 10 glomeruli in renal biopsies.
- Same induction therapy based on Kidney Disease Improving Global Outcomes (KDIGO) guidelines.
- Same maintenance therapy after remission.

### Exclusion criteria

- Previous administration of any immunosuppressive treatment for any reason.
- Clinical or laboratory manifestations of other systemic diseases, such as anti-glomerular basement membrane disease or Lupus nephritis.
- Patients with recent infection (less than 3 months).
- Patients not compliant to treatment regulations.
- Lost to follow-up.

### Assessment of kidney biopsies

Renal biopsies were evaluated by both a pathologist and a nephrologist, who were not aware of the patients’ clinical and laboratory manifestations. All kidney biopsies were classified according to Berden classification system and to Renal Risk Score, as described below:

- Berden classification as focal (≥ 50% normal < 50% injured glomeruli), crescentic (≥ 50% active crescents), mixed (≥ 50% injured glomeruli, < 50% crescents, < 50% global sclerosis) and sclerotic (≥ 50% global sclerosis).
- Renal Risk Score classification system: Three parameters were estimated for the Renal Risk Score, the proportion of normal glomeruli, severity of interstitial fibrosis/tubular atrophy and eGFR. Thus, for > 25%, 10–25%, or < 10% normal glomeruli, Renal Risk Score was graded by assigning 0, 4 or 6 points, respectively; for IFTA ≤ 25% or > 25%, Renal Risk Score was graded by assigning 0 or 2 points, respectively, and for eGFR > 15 ml/min or ≤ 15 ml/min, the system was graded by assigning 0 or 3 points, respectively. The resulting Renal Risk Score classified patients into Low risk group = 0 points, Medium risk group = 2–7 points and High risk group = 8–11 points.

In cases where there was disagreement between the pathologist and the nephrologist, biopsies were re-assessed by a third investigator, who was not aware of the previous results.

### Definitions

The eGFR was calculated using the “2021 CKD-EPI Creatinine equation”.

Standard treatment protocol for all included patients was:

*Induction treatment:* Intravenous pulses of cyclophosphamide in combination with intravenous methylprednisolone followed by oral corticosteroids with fast tapering according to the department protocol. Plasma-exchange was performed when indicated according to “KDIGO 2021 CLINICAL PRACTICE GUIDELINE FOR THE MANAGEMENT OF GLOMERULAR DISEASES”. Plasma exchange was applied in patients with serum creatinine levels (Scr > 5.7 mg/d), patients requiring dialysis or with rapidly increasing SCr, and in patients with diffuse alveolar hemorrhage.

*Maintenance treatment:* Steroids were gradually tapered by month 6 of follow up; Azathioprine 100 mg/d for at least 24 months.

The first 6 months following diagnosis (T0–T6) was regarded as the acute phase, while the period until 60 months of follow up (T60) was considered the chronic phase of the disease.

Relapse of AAV/GN was defined as the re-occurrence of clinical or laboratory evidence following remission according to KDIGO 2021 and treated with the same protocol as the initial diagnosis.

The Birmingham Vasculitis Activity Score (BVAS) score was calculated as described in Supplementary Table 1.

### Outcome

Primary end points: 1. ESKD at time points T3, T6 and T60 and the predictive value of Berden and Renal Risk Score scores. 2. Correlation of eGFR at T0, T3, T6 and T60 with calculated Berden and Renal Risk Score.

### Statistical analysis

Statistical analysis was performed by using the IBM Statistical Package for Social Sciences (SPSS) v27 for Windows. All continuous variables were estimated by the Shapiro–Wilk or Kolmogorov–Smirnov tests which were applied to assess the normality of the data distribution. Data from normally and non-normally distributed variables were expressed as Mean ± Standard Deviation and Median, and Interquartile Range, respectively. Differences between two independent variables were estimated by the Mann–Whitney *U* test, while changes during follow up were estimated by the Wilcoxon test, and the *p* values were subsequently adjusted by the Bonferroni correction. Odds Ratio (OR) and Receiver Operating Characteristics (ROC) curves were applied to estimate the incidence of the histopathology models in renal function, and the Kaplan–Meier test was performed to estimate renal and patient survival and relapse possibility. *p* Values of < 0.05 (two-tailed) were considered statistically significant for all comparisons.

## Results

### Clinical and laboratory findings at T0

One hundred and eight patients (*n* = 108), newly diagnosed with AAV/GN were initially assessed for study eligibility. Fourteen were excluded, including 7 with progressive disease requiring treatment start prior to kidney biopsy, 2 showing an association with anti-glomerular basement membrane disease, 3 with recent infections and 2 who moved and were lost to follow up. Supplementary Table 1 summarizes the baseline clinical characteristics of the enrolled patients.

### Renal histology

According to Berden classification, 24 (25.5%) were considered Focal, 35 (37.2%) Crescentic, 21 (22.3%) Mixed and 14 (14.9%) Sclerotic. The median levels of calculated Renal Risk Score was 6 (4.5), and based on Renal Risk Score, patients were classified as low, medium and high risk, 8 (8.6%), 46 (49.5%) and 39 (41.9%), respectively.

### Outcome of renal function

**Fig. 1:**
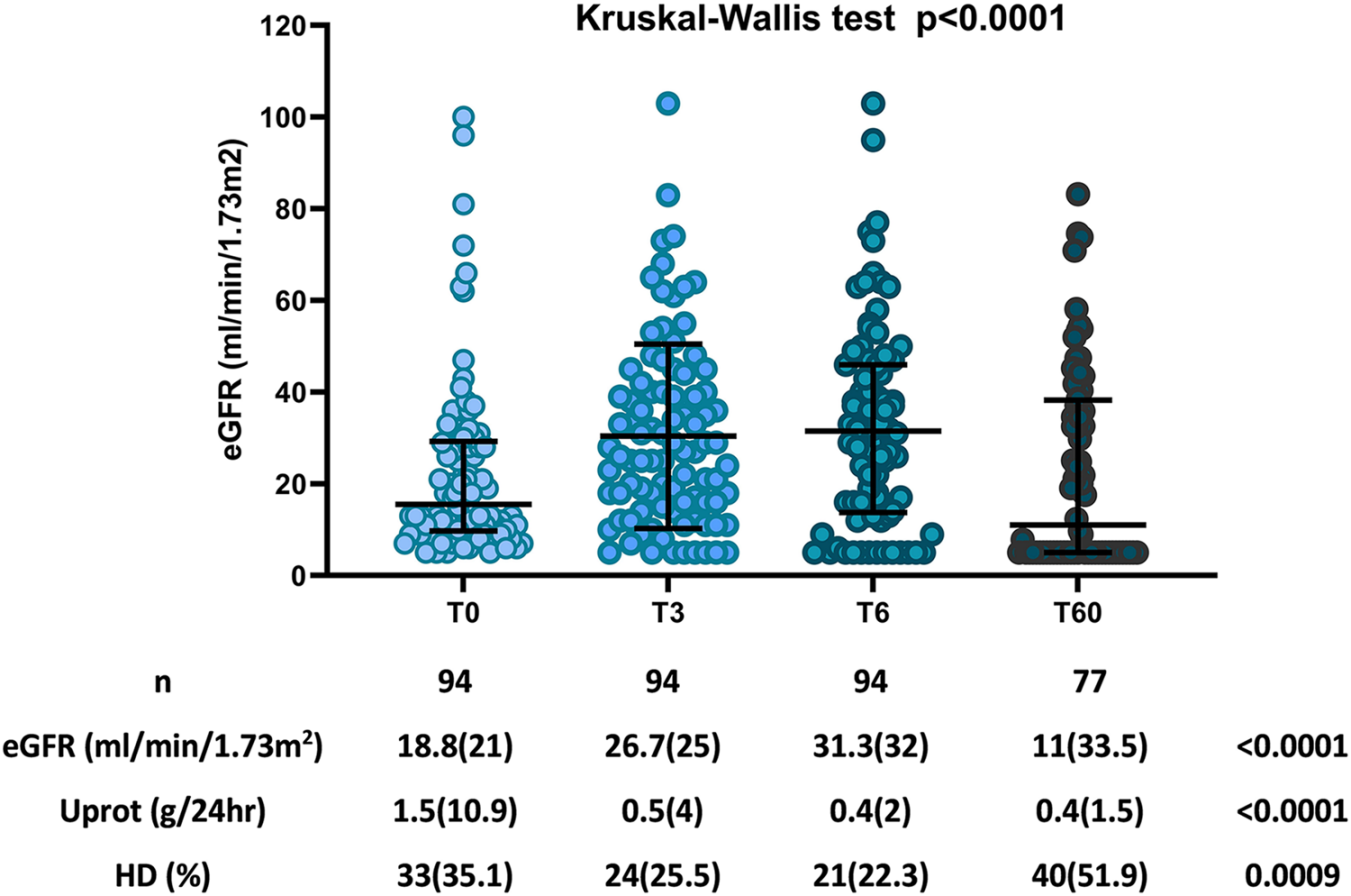
The upper panel of the figure depicts eGFR changes during follow up. In the lower panel, changes in eGFR, Uprot and need for HD are shown

Figure 1 depicts changes in renal function during follow up and changes in eGFR and proteinuria levels, as well as the proportion of patients who needed hemodialysis (HD) during the follow-up period.

### Correlation between Berden classification score and renal function outcome

Outcome of kidney function, as estimated by eGFR, and need for HD and patient survival at T0, T3, T6 and T60 were correlated to the Berden classification score.

Differences in eGFR at T0, T3, T6 and T60 between histopathology groups based on Berden risk score or Renal Risk Score, as well as changes in renal function during the acute (T6) and chronic (T60) follow-up period, are reported in Table 1.

**Table 1:** Differences in renal function outcome during follow up according to Berden classification and RRS, changes in renal function outcome and need for HD during the acute and chronic phase of the disease

Berden	*n*	eGFR (mL/min/1.73 m^2^)				*p *(T0–T6)	*p* (T0–T60)	No. of patients needing HD			
		T0	T3	T6	T60			T0	T3	T6	T60
Focal	24	18.3 (18)	27.6 (28)	31.1 (46)	19.1 (42.4)	0.08	0.06	4 (16.7%)	3 (12.5%)	4 (16.7%)	9 (47.4%)
Crescentic	35	12.8 (21)	21.7 (27)	25.3 (31)	12.3 (35)	0.01	0.03	18 (51.4%)	13 (37.1%)	10 (28.6%)	13 (48%)
Mixed	21	14.5 (18)	22.5 (18)	25.8 (33)	7.8 (29.5)	0.001	0.005	4 (19%)	2 (9.5%)	1 (4.8%)	8 (50%)
Sclerotic	14	13 (24)	18.8 (31)	16.6 (34)	9 (38.5)	0.38	0.38	7 (50%)	6 (42.9%)	6 (42.9%)	6 (54%)
*p*		0.14	0.03	0.24	0.94			0.01	0.02	0.03	0.98

RRS	*n*	eGFR (mL/min/1.73 m^2^)				*p* (T0–T6)	*p* (T0–T60)	No. of patients needing HD			
		T0	T3	T6	T60			T0	T3	T6	T60
Low	8	37.5 (56)	53 (37)	50 (23)	45.5 (22)	0.31	0.05	1 (12.5%)	0	0	0
Medium	47	23.5 (20)	35.1 (23)	37.5 (23)	32.6 (41.3)	< 0.0001	< 0.0001	7 (15.2%)	3 (6.5%)	3 (6.5%)	12 (31.6%)
High	39	10.6 (7)	15.4 (16)	14.5 (23)	5.7 (15)	0.05	0.002	24 (61.5%)	20 (51.3%)	17 (43.6%)	23 (69.7%)
*p*		< 0.0001	< 0.0001	< 0.0001	0.001			< 0.0001	< 0.0001	< 0.0001	< 0.0001

All data are presented as median (interquartile range—IQR)

Based on the results shown in Table 1, there were significant differences in eGFR levels between patients with Focal, Crescentic, Mixed and Sclerotic lesions (Berden classification score), but this was observed only at time point T3. During follow up, the most important changes in eGFR occurred in the Crescentic and Mixed groups, which demonstrated significant improvement at the end of the acute phase, i.e., T6, followed by declining renal function 5 years later, at T60.

There were also significant differences in the number of patients requiring HD according to Berden classification score, as described in Table 1. These differences however were prominent at presentation and during the acute phase, but disappeared at the end of follow up. Very interestingly, patients classified as Focal, showed a benign course from T0 to T6, with severe deterioration thereafter, patients classified as Crescentic and Mixed types had a similar course, with improvement from T0 to T6, followed by significant deterioration, and finally, patients classified as Sclerotic showed an unfavorable outcome early in the course of the disease. At time point T60 there were no significant differences between the Berden classification groups.

### Correlation between ANCA relative risk score and renal function outcome

The eGFR differences between groups, with Low, Medium and High Renal Risk Score were significant at all time points, i.e., T0, T3, T6 and T60 as depicted in Table 1. Interestingly, Low Renal Risk Score patients had improved renal function during follow up, from 37.5 (56) mL/min/1.73 m^2^ at T0 to 50 (23) mL/min/1.73 m^2^ at T6 and remained stable thereafter, until the end of follow up. Medium Renal Risk Score showed a temporary improvement; eGFR increased during the acute phase, from 23.5 (20) to 37.5 (23) min/1.73 m^2^ at T0 and T6, respectively, *p* < 0.0001 and, remained relatively stable, 32.6 (41.3) min/1.73 m^2^ until T60. In contrast, High Renal Risk Score patients had a completely different progression, they started with a compromised renal function at T0, 10.6 (7) min/1.73 m^2^, slightly improved to 14.5 (23) at T6, and progressed to 5.7 (15) at T60.

The number of patients requiring HD during follow up, according to the Renal Risk Score, is reported in Table 1. Again, the percentage of patients needing HD at all time points showed significant differences between Renal Risk Score groups. At the end of the acute phase, i.e., T6, this proportion ranged from 0% for the Low risk, to 6.5% and 43.6% for the Medium and High risk groups, respectively, *p* < 0.0001. At the end of follow up, i.e., T60, this difference became even more prominent from 0% to 31.6% and 69.7% at T0, T6 and T60, respectively (*p* < 0.0001).

### Sensitivity and specificity to predict dialysis dependence

**Fig. 2:**
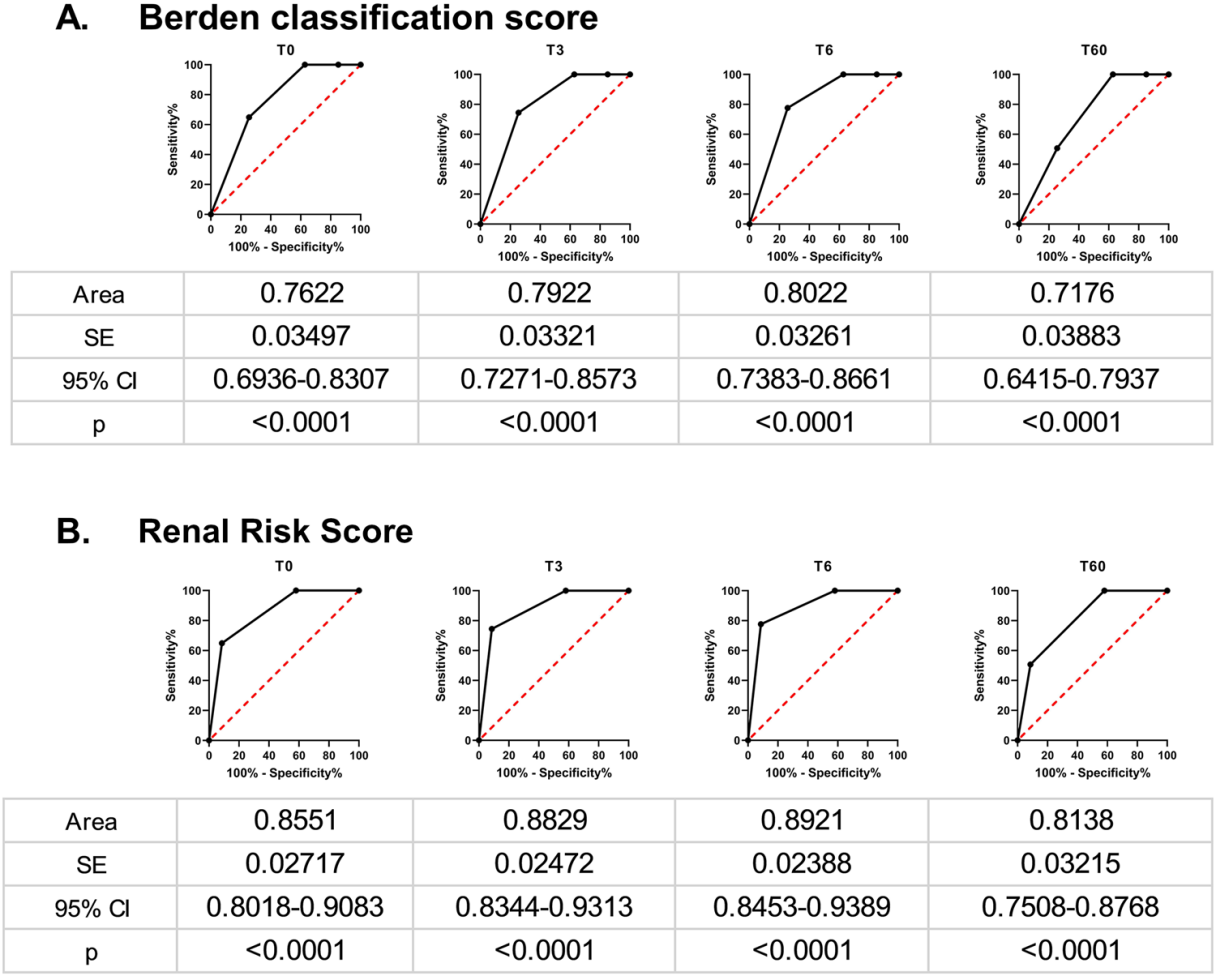
ROC curves for patients requiring HD at different time-points. **A** Berden classification score and **B** Renal Risk Score at time points T0, T3, T6 and T60

Sensitivity and specificity of the two classification systems to predict severe deterioration of renal function as estimated by ROC curves, are depicted in Fig. 2. Both scores demonstrated a significant impact on renal function, although Renal Risk Score seemed to be superior compared to Berden classification at all time-points.

### Kidney and patient survival

At the end of the follow up period, 40/94 (42.6%) patients progressed to ESKD, 26/94 (27.7%) had at least one relapse, and 4/94(4.3%) patients did not survive.

**Fig. 3:**
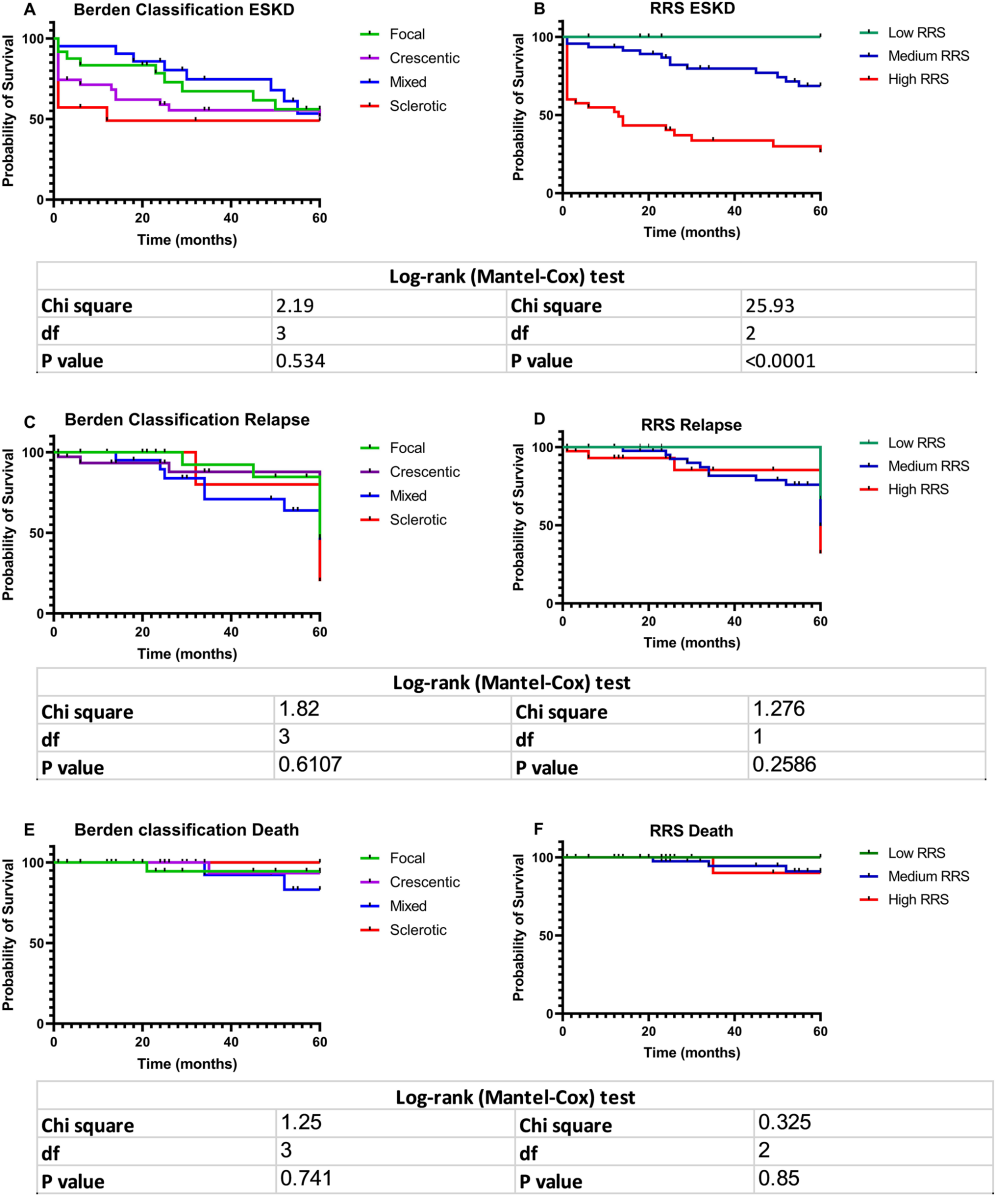
Kaplan–Meier curves of renal survival (**A**, **B**) relapse rate (**C**, **D**) and death (**E**, **F**) according to Berden and Renal Risk Score

As shown in Fig. 3, the Kaplan–Meier curves demonstrate the ability of the Renal Risk Score but not of the Berden classification system to predict ESKD. Relapses and death during the acute and long term follow-up period could not be predicted by either of the two systems.

## Discussion

In spite of extended research on AAV/GN, and despite the clarification of pathogenic mechanisms and immune pathways implicated, the diagnosis of the disease still relies on kidney biopsy findings [1, 12–15]. Moreover, at the moment, there are no reliable biomarkers which can determine treatment and predict outcome of renal function in these patients [1]. Shortly after the initial description of the disease, investigators admitted that treatment should be administered to all patients, regardless of renal dysfunction at presentation and severity of renal pathology [16–18]. Variability of pathology in AAV/GN, which includes coexisting active and chronic lesions, such as necrotic vasculitis, crescents, lymphoid infiltration, secondary focal sclerosis and interstitial fibrosis, highlights the need to describe and establish novel histopathologic classification models, which can become useful tools in the prognosis of AAV/GN. For this purpose, two models have recently been adopted, i.e., the Berden Classification and ANCA Renal Risk Score [6, 8].

In the present study we compared the predictive ability of these two highly promising and extensively used models, in terms of changes in eGFR, possibility of relapse and progression to ESKD during short- and long-term follow up. Both classification systems were able to discriminate patients who will have declining renal function or patients who will reach ESKD during the acute phase of the disease, however, the predictive ability of the Berden score was eliminated for the long term outcome, including both eGFR levels, and the percentage of patients reaching ESKD. Apparently, our results demonstrated similar results of the two classification systems in the short term, but showed clear superiority of the Renal Risk Score in predicting long-term outcome of renal function.

How can this be explained? Both classification systems evaluate changes in glomeruli, and take into account the percentage of normal glomeruli. The proportion of normal glomeruli in renal biopsy, i.e., glomeruli with no global or focal sclerosis and no crescents or necrosis, has indeed shown the best predictive ability for renal function outcome, up to 12 months of follow up [16]. Although the investigators’ interest was initially focused on active lesions in renal biopsy, namely cellular crescents and necrosis, they soon recognized the importance of the remaining unaffected glomeruli, as these could be protected by treatment and predict renal function [19].

However, there are few distinct differences between the two classification systems. Apart from normal glomeruli, the Berden classification system estimates the proportion of crescent formation and of globally sclerosed glomeruli. Furthermore, it excludes tubulointerstitial pathology and clinical presentation. The Renal Risk Score system emphasizes the proportion of normal glomeruli, without discriminating global sclerosis or crescents, but it shifts attention to the tubulointerstitial compartment, and accounts for IFTA lesions and renal function at time of diagnosis [5, 6].

The importance of renal interstitial fibrosis has been mainly assessed in a number of chronic glomerular diseases [20–22]. In these cases, interstitial fibrosis and tubular atrophy seem to be the main factors contributing to the progressive deterioration of renal function [23], and therefore, severity of IFTA has been extensively considered the main parameter predicting renal function outcome in glomerulopathies, such as membranous nephropathy, IgA nephropathy, and focal segmental glomerulosclerosis [13, 20–22]. Nevertheless, there are not enough studies regarding the role of IFTA in predicting renal function outcome in AAV/GN. The natural course of AAV/GN seems to be the main reason for this, as the disease progresses quickly, not allowing chronic lesions, such as IFTA to develop and participate in renal function outcome. During the last few years, and likely due to the amelioration of therapeutic strategies which resulted in better disease outcome, the importance of interstitial and tubular lesions in disease outcome have been progressively recognized. Thus, chronic lesions in kidney biopsy have been recognized as an independent factor which predicts renal function and response to treatment in AAV/GN [24], while urinary excretion of molecules associated with interstitial fibrosis, such as IL-6, MCP-1 and recently, Dickkopf-related protein 3, PRO-C6 and C3M have been shown to be biomarkers of disease outcome [25–27].

Inferiority of the Berden classification system in predicting long term outcome of renal function is not a new finding. Previous studies showed that it could not offer further assistance in describing disease severity and outcome, from distinct description of histologic findings [28]. Additionally, Berden Classification system provides a clear distinction for the focal and sclerotic classes, yet does not seem to effectively distinguish between crescentic and mixed classes [9, 29, 30].

In our study, we used only the Cyclophosphamide-Azathioprine regimen, and our patients received induction therapy with cyclophosphamide in combination with intravenous/oral corticosteroids, and maintenance therapy with corticosteroids and Azathioprine. This could be a limitation of the study, as we excluded patents who received Rituximab, and therefore, we do not have data regarding protocols including Rituximab administration.

Although the prognostic value for long-term renal outcome was not confirmed in our study, the fact that even in patients with sclerotic class, eGFR was significantly higher at T3 and T6, indicates that all patients, regardless of the percentage or the type of glomeruli injury, should receive treatment.

## Conclusions

Histopathologic lesions can provide useful information about the possibility of ESKD development in patients with AAV/GN. Both scores are effective in predicting the short-term outcome, while only Renal Risk Score, that takes into account the number of normal glomeruli, eGFR and both interstitial fibrosis and tubular atrophy, proved to sufficiently predict long-term renal function in AAV/GN. Certain differences in the Renal Risk Score, including the addition of IFTA findings and initial eGFR, are the main parameters resulting in the superiority of the present classification system in long-term follow up.

### Supplementary Information

Below is the link to the electronic supplementary material.

Supplementary file1 (DOCX 79 KB)

## Data Availability

Data will be available upon request to the corresponding author.
