# Supplementary material for: The prognostic value of two histopathologic classification models of ANCA-associated glomerulonephritis: a prospective study
Source: J Nephrol. 2024 Feb 12;37(4):941–50. doi: 10.1007/s40620-023-01855-x (PMC11239783; doi:10.1007/s40620-023-01855-x)

**Supplementary Material**

**Suppl. Table 1.** Clinical and biochemical Baseline Characteristics

| **Patients** | **Total n=94** |
| --- | --- |
| Age (years) | 60.05 (18-82) |
| Male/Female | 36/58 |
| ANCA type (%)  (MPO/PR3/ANCA neg) | MPO: 41 (43.6) |
|  | PR3: 35 (37.2) |
|  | ANCA negative: 18 (19.1) |
| BVAS score | 15.6±3.6 |
| Renal involvement (%) | 94 (100) |
| eGFR at T0 (ml/min/1.73m^2^) | 18.8(21) |
| Serum Creatinine (mg/dl) | 3.2(1.8) |
| Serum Urea (mg/dl) | 105(68) |
| Urine protein (g/24hr) | 1.1(1.4) |
| Dialysis Dependence (%) | 33 (35.1) |
| Upper respiratory system (%) | 18(19.1) |
| Lower respiratory system (%) | 42(44.7) |
| Rash (%) | 20(21.3) |
| Arthritis (%) | 32(34) |
| Hypertension (%) | 41(43.6) |

**Suppl. Figure 1.** Classification of patients according to Berden (A) and Renal risk score (RRS) (B)


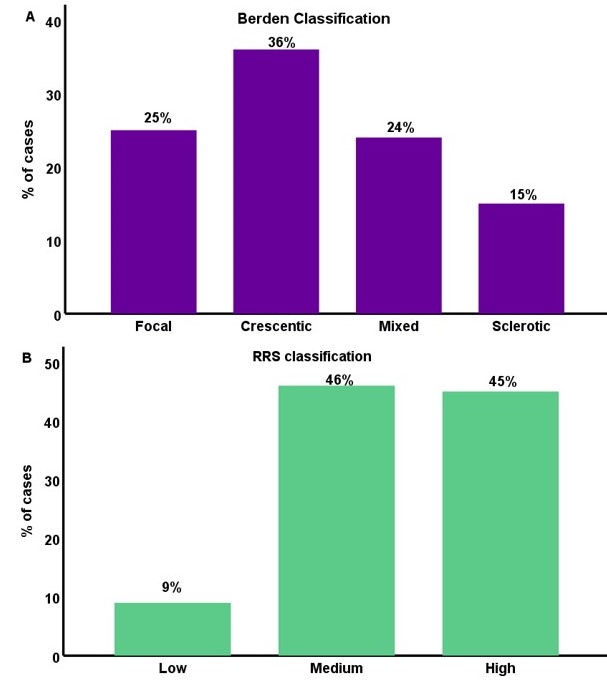

Supplement: Supplementary file 1 — Supplementary file1 (DOCX 79 KB) [file 40620_2023_1855_MOESM1_ESM.docx]
